# Surgeon-General William Watson

**Published:** 1912-06-22

**Authors:** 


### Obituary.

Surgeon-General William Watson, M.D., of the Indian Medical Service, has died at the age of eighty. After graduating at Aberdeen University, he entered the service of the East India Company as assistant surgeon. He served through the Mutiny.

